# Combining PLGA Scaffold and MSCs for Brain Tissue Engineering: A Potential Tool for Treatment of Brain Injury

**DOI:** 10.1155/2018/5024175

**Published:** 2018-08-05

**Authors:** Ling Zhou, Jiangyi Tu, Guangbi Fang, Li Deng, Xiaoqing Gao, Kan Guo, Jiming Kong, Jing Lv, Weikang Guan, Chaoxian Yang

**Affiliations:** ^1^Department of Endocrinology, The Affiliated Hospital, Southwest Medical University, Taiping Street, Luzhou 646000, China; ^2^Department of Anatomy, Southwest Medical University, Zhongshan Road, Luzhou 646000, China; ^3^Department of Neurobiology, Preclinical Medicine Research Center, Southwest Medical University, Zhongshan Road, Luzhou 646000, China; ^4^Department of Human Anatomy and Cell Science, College of Medicine, University of Manitoba, Winnipeg, MB, Canada

## Abstract

Nerve tissue engineering is an important strategy for the treatment of brain injuries. Mesenchymal stem cell (MSC) transplantation has been proven to be able to promote repair and functional recovery of brain damage, and poly (lactic-co-glycolic acid) (PLGA) has also been found to have the capability of bearing cells. In the present study, to observe the ability of PLGA scaffold in supporting the adherent growth of MSCs and neurons in vivo and vitro and to assess the effects of PLGA scaffold on proliferation and neural differentiation of MSCs, this study undertakes the following steps. First, MSCs and neurons were cultured and labeled with green fluorescent protein (GFP) or otherwise identified and the PLGA scaffold was synthesized. Next, MSCs and neurons were inoculated on PLGA scaffolds and their adhesion rates were investigated and the proliferation of MSCs was evaluated by using MTT assay. After MSCs were induced by a neural induction medium, the morphological change and neural differentiation of MSCs were detected using scanning electron microscopy (SEM) and immunocytochemistry, respectively. Finally, cell migration and adhesion in the PLGA scaffold in vivo were examined by immunohistochemistry, nuclear staining, and SEM. The experimental results demonstrated that PLGA did not interfere with the proliferation and neural differentiation of MSCs and that MSCs and neuron could grow and migrate in PLGA scaffold. These data suggest that the MSC-PLGA complex may be used as tissue engineering material for brain injuries.

## 1. Introduction

In recent years, the development of tissue engineering has provided a new strategy for the repair of tissue injuries. The core of tissue engineering is to construct new tissue substitutes composed of biological materials and cells for promoting the recovery and maintenance of biological functions [1, 2]. Biological materials not only offer three-dimensional space for cell adhesion, growth, and migration but also form adjustable microenvironments for the nutrition obtainment and waste excretion of cells [3]. Biological materials used for neural tissue engineering can be mainly divided into 5 categories: artificial synthetic nonbiodegradable materials, nondegradable composite ducts, natural biological materials, biodegradable composites, and biodegradable polymer materials.

Poly (lactic-co-glycolic acid) (PLGA) is one biodegradable polymer material and the degradation time of PLGA can be adjusted simply by altering the ratio of lactic acid and glycolic acid in its copolymer for particular applications. PLGA with a ratio of 75 : 25 of PLA : PGA showed great stability in body fluids (pH 7.2) with an optimum degradation rate (9% to 12% or so), and axons could regenerate into the implanted PLGA scaffolds in rats subjected to thoracic spinal cord transection injury [4]. Mesenchymal stem cells (MSCs) could differentiate into neuron-like cells under specific culture conditions and had some electrophysiological properties of neurons [5–7], which makes them a kind of seed cells for the treatment of nerve tissue injuries. The aim of this study is to evaluate whether the MSC-PLGA scaffold complex is a potential tool for the treatment of brain injuries.

## 2. Materials and Methods

### 2.1. Preparation and Labeling of MSCs

Two-month-old adult and 1-day-old newborn Sprague Dawley (SD) rats (Animal House Center, Southwest Medical University) were used in this study. The procedure to use the animals was in accordance with the Guidance Suggestions for the Care and Use of Laboratory Animals formulated by the Ministry of Science and Technology of China. Bone marrow was obtained from femoral marrow cavities of 2-month-old rats. The MSCs were isolated and purified from bone marrow by density gradient centrifugation and adherent culture methods, and they were cultured by using alpha-minimum essential medium (*α*-MEM) (HyClone) supplemented with 10% FBS, 100 U/ml penicillin, and 100 mg/ml streptomycin (Gibco). When the cultured cells became confluent, they were resuspended and subcultured.

To facilitate the observation of the growth and migration of MSCs in the PLGA scaffold or a brain, MSCs were labeled with green fluorescent protein (GFP) as previously described [8]. Briefly, we amplified the previously frozen adenovirus (pAdEasy-1-pAdTrack cytomegalovirus) that contained the GFP gene. When the third passage MSCs grew to 70–80% confluence, the adenovirus solution was added, which was followed by incubation for 2 days in an incubator (37°C, 5% CO2), and MSCs showed green fluorescence under fluorescent microscopy. Then the cells were harvested for follow-up experiments.

### 2.2. Preparation and Identification of Neurons

Cortical neurons were prepared from 1-day-old newborn rats as previously described [9]. Newborn rats were decapitated, and cerebral cortexes were transferred to PBS. After the removal of the meninges and blood vessels, tissues were cut into small pieces, followed by incubation in 0.25% trypsin-EDTA solution (Beyotime) at 37°C for 20 minutes. Then LG-DMEM with 20% fetal bovine serum was added to terminate the incubation. After centrifugation at 800 rpm for 10 minutes, the cells were collected for follow-up experiments.

Cortical neurons were dispersed with a neuronal medium (Sciencell) with 1% (vol/vol) neuronal growth supplement (Sciencell), and then they were seeded at a density of 5 × 10^5^/ml onto coverslips precoated with poly-L-lysine in 6-well plates (Corning). The medium was replaced once every 3 days, and after 7 days, the cells were fixed with 4% formaldehyde and used for the identification of neurons by immunocytochemistry.

### 2.3. Fabrication of PLGA Scaffold

The PLGA was synthesized by the room temperature molding/particle leaching method as previous described [8]. In short, 75% lactic acid and 25% glycolic acid were dissolved in dichloromethane and blended with sieved sodium chloride particles ranging from 80 to 120 *μ*m. The mixture was poured in molds to form discs (5 cm × 5 cm × 0.2 cm). After molding for 24 hours under pressure, the discs were taken out and immersed in deionized water to release the sodium chloride particles and the scaffolds were desiccated and kept in a vacuum plastic bag before use.

### 2.4. Cell Adhesion on PLGA Scaffold

The PLGA scaffold was cut into pieces (1 cm × 1 cm × 0.2 cm). The latter were dipped in 75% alcohol for 2 hours and then washed 3 times with sterile water and dried in clean bench; after that, the sterile PLGA scaffolds were placed in a 24-well culture plate. MSCs and neurons were seeded on the scaffolds at a density of 1 × 10^5^ cells per scaffold in the corresponding medium under standard cell culture conditions. After 3 days, the culture mediums were removed and the cells on the scaffolds and wells were collected by trypsin digestion and counted as *n*_PLGA_ and *n*_well_, respectively. The adhesion rates of MSCs and neurons on scaffolds were calculated as follows:

(1) $$\begin{array}{c} r=\frac{n_{PLGA}}{n_{PLGA}+n_{well}}\times 100\%. \end{array}$$

### 2.5. MTT Assay

The effect of the PLGA on the proliferation of MSCs in vitro was assessed by using 3-(4,5-dimethylthiazol-2-yl)-2,5-diphenyl tetrazolium bromide (MTT) assay based on the instruction manual of the MTT Cell Proliferation and Cytotoxicity Assay Kit (Beyotime). Briefly, MSCs were seeded on PLGA scaffolds at a seeding concentration of 1 × 10^5^ cells per scaffold (1 cm × 1 cm × 0.2 cm) per well of the 24-well culture plate and cultured for 48 hours. The control group was identically processed, except that the PLGA scaffolds were omitted. The culture medium was replaced with fresh medium for 24 hours. Then the MTT reaction solution was added to each well, and next, the plate was incubated for 4 hours at 37°C. After the mediums of all wells were removed, formazan dissolving solution was added into each well and incubated at 37°C for 4 hours. The supernatants of all wells were transferred into a 96-well culture plate, and the absorbance of each well was measured at 570 nm by a microplate reader.

### 2.6. Neuronal Induction of MSCs

MSCs were seeded on coverslips and PLGA scaffolds in a 24-well culture plate in *α*-MEM supplemented with 10% FBS (HyClone). After 3 days, the medium was replaced with a preinduction solution composed of *α*-MEM with 10% FBS and 1 mmol/l *β*-mercaptoethanol (*β*-ME) for 24 hours. This was followed by a neuronal induction medium that consisted of *α*-MEM with 1 mmol/l *β*-ME, 2% dimethyl sulfoxide (DMSO), and 1 *μ*mol/l all-trans retinoic acid (RA) (Sigma) for 6 hours. Five coverslips and 5 PLGA scaffolds were fixed with 4% paraformaldehyde (PFA) in phosphate-buffered saline, and the scaffolds were cut into 15 *μ*m sections. The coverslips and sections were used to observe the effect of the PLGA scaffold on the differentiation of MSCs via immunocytochemistry. Five scaffolds were applied for transplantation and 5 scaffolds fixed with 4% glutaraldehyde were utilized for scanning electron microscopy (SEM).

### 2.7. PLGA Scaffold Transplantation

The traumatic brain injury (TBI) model was generated as previously described [10]. In brief, a 2-month-old rat was anesthetized with 1% pentobarbital sodium (40 mg/kg) via intraperitoneal administration and then fixed on a stereotaxic frame (Stoelting) in the prone position. Following the scalp incision, a piece of right parietal bone was removed by drilling. A dual incision was made to expose the forebrain, and a defect area (3 mm × 3 mm × 2 mm) was created with a scalpel in the brain. The PLGA scaffold or MSC-PLGA scaffold complex was inserted in the brain, and the wound was sutured. On day 14 after TBI, rats (5/group) were killed by deep anesthesia and their brains were removed and then PLGA scaffolds and MSC-PLGA scaffold complex were taken for SEM. The other rats were perfused transcardially with 0.9% saline followed by ice-cold 4% PFA. The brains were taken out and postfixed in 4% PFA for 24 h. Then they were dehydrated with 30% sucrose solution, embedded in tissue-freezing medium, and cut into serial coronal sections (15 *μ*m thickness) with a freezing microtome (Leica) for nuclear staining and immunohistochemical staining.

### 2.8. Scanning Electron Microscopy

Scanning electron microscopy (S-3400N, Hitachi, Japan) was used to observe the characteristics of the PLGA scaffold and the morphologies of the cells attached to it. Prior to imaging, cells that were cultured or grown on the scaffolds were fixed with 4% glutaraldehyde and dehydrated through a graded acetone series and then sputter coated with gold. Samples were examined at an accelerating voltage of 10 kV.

### 2.9. Immunohistochemistry

The slides of cells or frozen sections were treated with 0.3% Triton X-100 and then blocked with 8% goat serum. After being incubated with primary antibodies including *β*-tubulin (1 : 100 dilution, Abcam), microtubule-associated protein-2 (MAP2) (1 : 100 dilution, Abcam), or glial fibrillary acidic protein (GFAP) (1 : 200 dilution, Sigma) overnight at 4°C, the samples were treated with anti-rabbit/mouse IgG (Alexa Fluor® 488/Fluor 594 Conjugate) (1200 dilution; Cell Signaling Technology) for 30 min at 37°C and then stained with Hoechst dye. Negative controls were identically processed, except that the primary antibodies were omitted.

### 2.10. Statistical Analysis

All cell experiments in vitro were repeated three times and the analyses were performed using the SPSS 18.0 software for Windows. The data are presented as the means ± standard error (SE), and the statistical comparisons were performed using one-way ANOVA. A *P* value < 0.05 was considered statistically significant.

## 3. Results

### 3.1. Morphologic Characteristics of Cultured Cells

**Figure 1:**
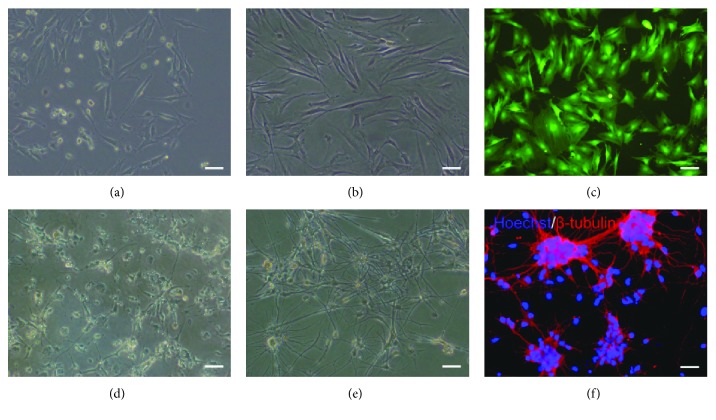
Morphologic characteristics of MSCs and neurons. (a) The primary MSCs were cultured for 4 days. (b) The 3rd-passage MSCs were cultured for 2 days. (c) The MSCs infected with adenovirus for 2 days were lighted green fluorescence. The primary neurons were cultured for (d) 3 days and (e) 7days. (f) The identification of neurons by immunostaining with *β*-tubulin. Bar = 50 *μ*m.

The primary MSCs began to adhere within 12 hours and presented round, polygon, or spindle shapes after 3-4 days (Figure 1(a)). The 3rd passage of MSCs displayed obvious uniformity (Figure 1(b)), and they were infected by the adenovirus-lighted green fluorescence under fluorescence microscope (Figure 1(c)). The primary cortical neurons showed fewer and shorter protuberances within 3 days (Figure 1(d)). Then many neurites appeared, which formed many neural networks on the seventh day (Figure 1(e)), and presented positive *β*-tubulin staining via immunocytochemistry (Figure 1(f)).

### 3.2. Attachment of MSCs and Neurons on PLGA Scaffolds

**Figure 2:**
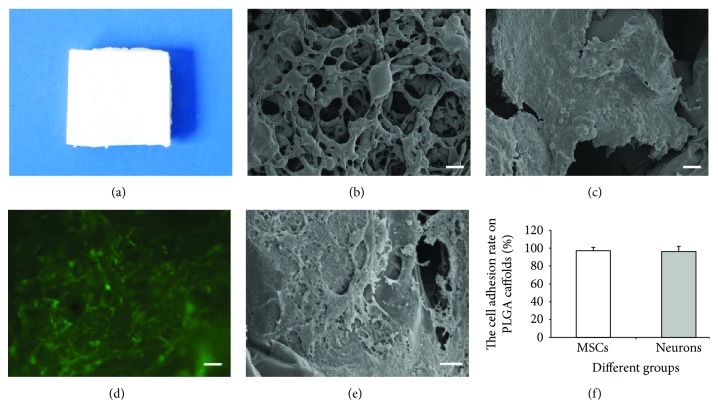
PLGA scaffold and cell adhesion. (a) PLGA scaffold. (b) SEM imaging of the PLGA scaffold. Bar = 100 *μ*m. (c) SEM imaging of the neurons on the PLGA scaffold. Bar = 10 *μ*m. (d) MSCs planted on the PLGA scaffold were lighted green fluorescence. Bar = 50 *μ*m. (e) SEM imaging of MSCs on the PLGA scaffold. Bar = 10 *μ*m. (f) The adhesion rates of MSCs and neurons on the PLGA scaffold.

The volume of porosity of the PLGA scaffold approached 90% by using the liquid replacement approach, and the interior pores of the PLGA scaffold that were directly visualized by SEM were intercommunicated (Figures 2(a) and 2(b)). After culturing of the MSCs and neurons for 5 days, a large number of cells were found adhered and extended on the surface of the PLGA scaffold and the cells were flat and connected to each other (Figures 2(c)–2(e)). On the 3rd day after inoculation, the adhesion rates of MSCs and neurons on the PLGA scaffold were 97.4% and 96.5%, respectively, and there was no significant difference between the two groups (Figure 2(f)).

### 3.3. The Effect of PLGA Scaffold on Differentiation and Proliferation of MSCs In Vitro

**Figure 3:**
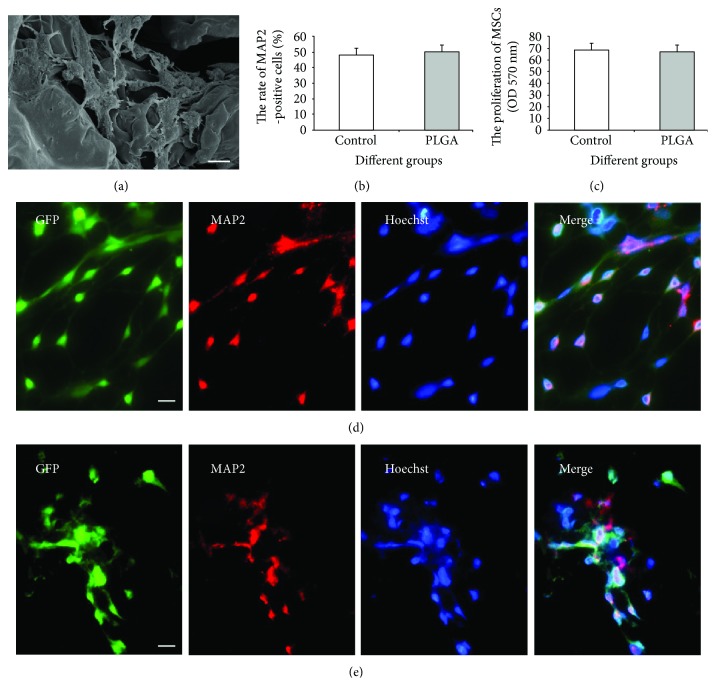
The effect of the PLGA scaffold on the differentiation and proliferation of MSCs in vitro. (a) SEM imaging of induced MSCs planted on the PLGA scaffold. Bar = 10 *μ*m. (b) The rate of MAP2-positive cells among the MSCs after neural induction. (c) The proliferation of MSCs on the coverslip and PLGA scaffold. The control group (d) and the PLGA scaffold group (e): green fluorescence showed MSCs in vitro. Neurons (MAP2 positive) were stained with red fluorescence. Yellow fluorescence showed the colocalization of green and red, thus indicating the differentiation of MSCs. Bar =50 *μ*m.

After induction, the MSCs presented neuron-like morphology with swollen cell bodies and long thin processes, and intercellular boundaries became manifested (Figure 3(a)). The induced MSCs could express the marker of neurons (MAP2), and the rate of both MAP2^+^ MSCs in the PLGA scaffold group was not significantly different compared with that in the control group (Figures 3(b), 3(d), and 3(e)). Furthermore, MTT assay was used to evaluate the proliferation of the MSCs cultured on the PLGA scaffold and the MSCs showed similar absorbance in the PLGA and control groups after incubation for 7 days (*P* < 0.05) (Figure 3(c)). These results suggest that the PLGA scaffold did not interfere with the proliferation and neuronal differentiation of MSCs in vitro.

### 3.4. The Structure of PLGA In Vivo

**Figure 4:**
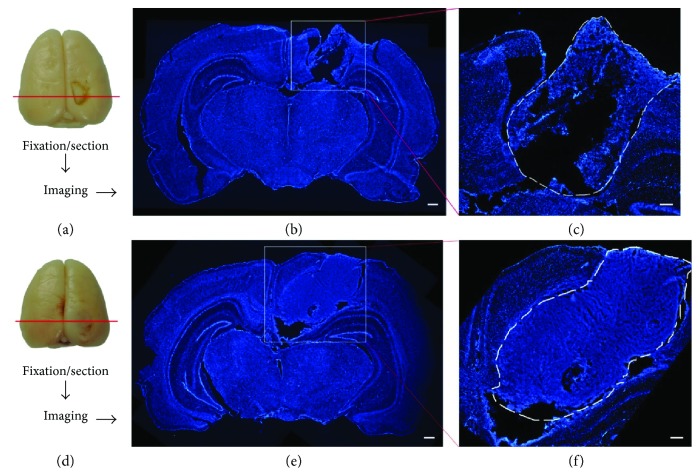
The structural change of the PLGA scaffold in brains with TBI. (a) Procedure to observe PLGA scaffold in the brain with TBI. (b) Nuclear staining shows the planted PLGA scaffold in the brain. Bar = 500 *μ*m. (c) The magnifying picture of the square frame in (b). The PLGA scaffold in the outlined region (dashed line). Bar = 200 *μ*m. (d) Procedure to observe the MSC-PLGA scaffold complex in the brain with TBI. (e) Nuclear staining shows the planted MSC-PLGA scaffold complex in the brain. Bar = 500 *μ*m. (f) The magnified picture of the square frame in (e). The MSC-PLGA scaffold complex in the outlined region (dashed line). Bar = 200 *μ*m.

The structure of the PLGA in brains was assessed by morphological observation with nuclear staining at 14 days after TBI. Under a microscope, the tissue organization of the transplanted PLGA scaffold was distinctive between the PLGA scaffold group (Figures 4(a)–4(c)) and the MSC-PLGA scaffold group (Figures 4(d)–4(f)). In the former, the tissue structure was looser and the interstitial space was larger, while a more compact structure and smaller spaces could be observed in the latter.

### 3.5. Cell Migration and Adhesion in PLGA Scaffold In Vivo

**Figure 5:**
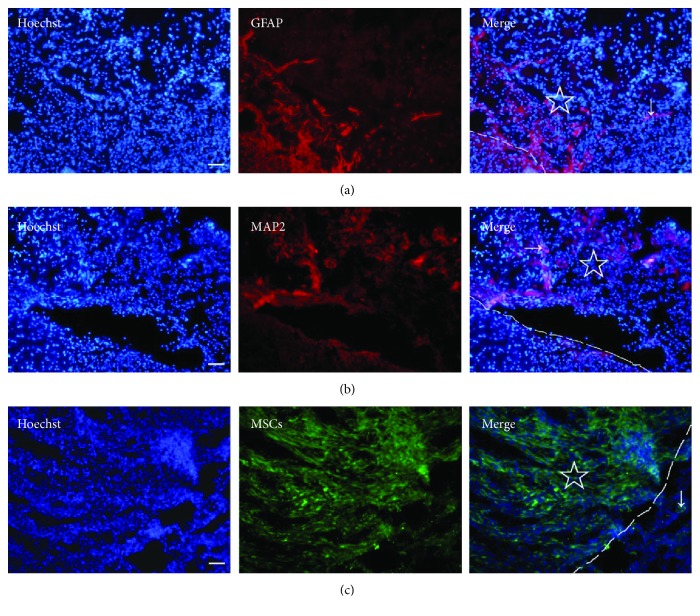
Cell migrations in the PLGA scaffold in the brain with TBI. (a) Astrocytes (arrow) stained with anti-GFAP (red) migrated in the PLGA scaffold. (b) Neurons (arrow) stained with anti-MAP2 (red) migrated in the PLGA scaffold. (c) MSCs (green, arrow) migrated out of the MSC-PLGA scaffold complex. “☆” shows the PLGA scaffold or the MSC-PLGA scaffold complex in the brain, and the dashed line indicates the boundary of the scaffold. Bar = 50 *μ*m.

**Figure 6:**
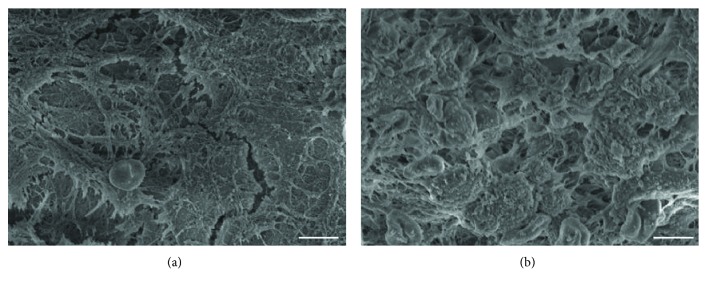
Cells' adhesion on the PLGA scaffold in the brain with TBI. (a) SEM imaging of cells' adhesion on the PLGA scaffold. (b) SEM imaging of cells' adhesion on the MSC-PLGA scaffold complex. Bar = 5 *μ*m.

After the transplantation of the PLGA scaffold into the brain at 14 days after TBI, some cells migrated into the PLGA, including glial cells (Figure 5(a)) and neurons (Figure 5(b)). When the MSC-PLGA scaffold complex was implanted in the brain, the MSCs could migrate out to the adjacent brain area (Figure 5(c)). In addition, we found that cells could adhere better on the MSC-PLGA scaffold complex than the PLGA scaffold (Figures 6(a) and 6(b)). The results suggest that the PLGA-mixed MSCs are more beneficial to cell adhesion and migration, and thus, the combination of the brain tissue and scaffold is closer.

## 4. Discussion

Compatibility between the biomaterial scaffold and seed cells is a core issue to construct engineered tissues and organs. The biomaterial scaffold as the cell carrier and exogenous graft should have a positive effect or no palpable side effect on cell growth and differentiation, and it should be accompanied by good biocompatibility and easy degradation in vivo after implantation [11, 12]. PLGA has been served as a drug carrier because of its biocompatibility and biodegradation [13, 14]. Seed cells derived from autologous tissue used to construct engineered tissue are the preferable choice. MSCs have received extensive attention because they can be obtained from autologous tissue and they were easily isolated, expanded in vitro, and further induced to differentiate into neuronal cells, adipocytes, osteoblasts, myocytes, and other cell types [15–18]. In this study, we found that PLGA did not interfere with the proliferation and neural differentiation of MSCs.

Cell adhesion and migration properties will influence the proliferation, differentiation, and function of cells [19, 20]. To achieve the function of the cell scaffold, the cell carrier should ensure good adhesion, growth, and reproduction of seed cells and host cells. In the present work, the advantages show that PLGA scaffolds are capable of supporting 3D growth for MSCs and neurons in vivo and in vitro and neural-induced MSCs can still adhere to the PLGA scaffold. Previous studies have shown that MSCs can secrete various extracellular matrixes, neurotrophic factors, and cell adhesion factors [21, 22] and the transplantation of MSCs can promote the repair of central nervous system injuries [23, 24]. We found that the transplantation of the MSC-PLGA complex made the impaired brain more complete than that of the simple PLGA scaffold. In summary, our results suggested that MSC-PLGA may be used as suitable graft for nerve tissue engineering, but its biological properties in vivo merit further study.

## Data Availability

The data used to support the findings of this study are available from the corresponding author upon request.
